# SUMOylation in *Giardia lamblia*: A Conserved Post-Translational Modification in One of the Earliest Divergent Eukaryotes

**DOI:** 10.3390/biom2030312

**Published:** 2012-07-25

**Authors:** Cecilia V. Vranych, María C. Merino, Nahuel Zamponi, María C. Touz, Andrea S. Rópolo

**Affiliations:** Instituto de Investigación Médica Mercedes y Martín Ferreyra, INIMEC- CONICET, Friuli 2434, Córdoba, Argentina; Email: cvranych@immf.uncor.edu (C.V.V.); mcmerino@immf.uncor.edu (M.C.M.); nzamponi@immf.uncor.edu (N.Z.); ctouz@immf.uncor.edu (M.C.T.)

**Keywords:** *Giardia lamblia*, SUMO, SUMOylation pathway, monoclonal antibody production, post-translational modifications

## Abstract

Post-translational modifications are able to regulate protein function and cellular processes in a rapid and reversible way. SUMOylation, the post-translational modification of proteins by the addition of SUMO, is a highly conserved process that seems to be present in modern cells. However, the mechanism of protein SUMOylation in earlier divergent eukaryotes, such as *Giardia lamblia*, is only starting to become apparent. In this work, we report the presence of a single SUMO gene encoding to SUMO protein in *Giardia*. Monoclonal antibodies against recombinant *Giardia* SUMO protein revealed the cytoplasmic localization of native SUMO in wild-type trophozoites. Moreover, the over-expression of SUMO protein showed a mainly cytoplasmic localization, though also neighboring the plasma membrane, flagella, and around and even inside the nuclei. Western blot assays revealed a number of SUMOylated proteins in a range between 20 and 120 kDa. The genes corresponding to putative enzymes involved in the SUMOylation pathway were also explored. Our results as a whole suggest that SUMOylation is a process conserved in the eukaryotic lineage, and that its study is significant for understanding the biology of this interesting parasite and the role of post-translational modification in its evolution.

## 1. Introduction

*Giardia lamblia* is one of the most prevalent parasitic protozoan in developing countries, causing an intestinal pathology known as giardiasis, which in many cases produces diarrhea and nutrient malabsorption in humans [1,2]. It has a simple life cycle with two major stages: infectious cysts and trophozoites [2], which have specific mechanisms enabling them to adapt to their environment [3]. These mechanisms involve the preferential expression of genes and proteins to allow parasite survival and the transmission of the pathology to susceptible hosts. Although its phylogenetic position in the eukaryotic lineage is controversial at the moment, *Giardia* is considered an early divergent eukaryote in evolution and possesses unusual features, such as the presence of two transcriptionally active diploid nuclei and the absence of mitochondria and peroxisome [4], which make this an attractive model to study the evolution of regulatory systems. 

Post-translational modifications are one of the most effective ways by which evolution has increased versatility in protein function, providing the cell with the flexibility to respond to a broad range of stimuli [5,6]. These modifications are essential and reversible mechanisms by which the functions, activities, and stabilities of preexisting proteins can be rapidly and specifically modulated, thereby controlling dynamic cellular processes [7]. Interaction with Small Ubiquitin-like Modifier (SUMO) is, in particular, one of the most complex, conserved, and interesting characteristic mechanisms of protein regulation in eukaryotes, with diverse targets and functions such as nuclear transportation, transcriptional regulation, maintenance of genome integrity, and signal transduction [6,8,9].

**Figure 1:**
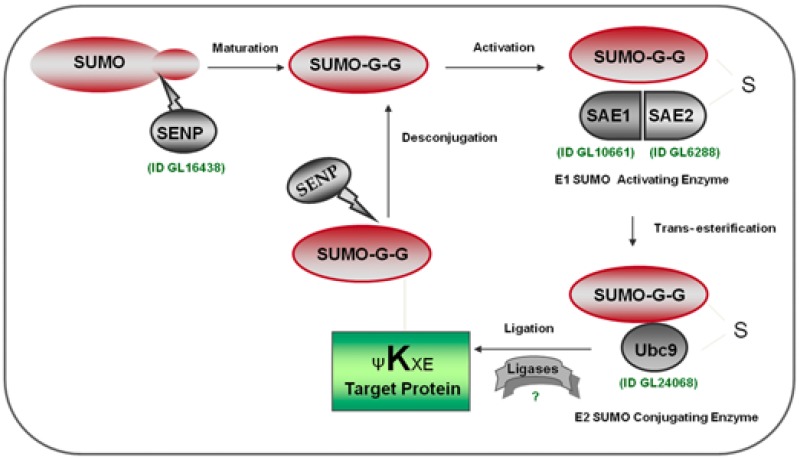
The SUMO conjugation pathway. SUMO is expressed as an inactive propeptide and is processed by a SUMO-specific protease (SENP) to expose the C-terminal GG, required by the SUMO conjugation to targets (maturation). Mature SUMO is activated by the SUMO activating enzyme (E1) and is transferred through a transesterification process to Ubc9 (E2). SUMO is next conjugated to the target lysine of a substrate, defined by the consensus motif ΨKXE. E3 ligase enzyme can facilitate this process. Specific proteases can remove SUMO from modified substrates maintaining the reserve of free SUMO. Gene ID corresponding to homologous *Giardia* enzymes involve in the SUMOylation process is depicted in green. Modified from [10].

SUMO belongs to the ubiquitin-like protein family (Ubl), displaying a three-dimensional structure similar to ubiquitin, although it shares only 18% identical amino acids and differs in the distribution of charged residues on the surface [5,8]. Like ubiquitin, SUMO is expressed as a precursor protein and requires a maturation process, by specific SUMO proteases (SENPs) (Figure 1), to expose the carboxy-terminal double-glycine motif (GG) required for conjugation to substrate proteins [10]. SUMO is covalently attached to target proteins, via an isopeptide bond between a C-terminal glycine of SUMO and a lysine residue within the consensus sequence defined by ψKXE (where ψ is a large hydrophobic amino acid, K is the lysine to which SUMO is conjugated, X is any amino acid, and E is glutamic acid residue) [8,11]. 

As an ubiquitination process, conjugation to SUMO involves an enzymatic cascade, which includes an E1-activating enzyme, an E2-conjugating enzyme, and sometimes the assistance of a ligase that increases the efficiency of transferring to substrate [12,13]. Unlike the ubiquitin E1 enzyme, which functions as a single subunit enzyme, the SUMO E1 enzyme consists of a heterodimer of two polypeptides known as SUMO Activation Enzyme 1 and 2 (SAE1 and SAE2) [5]. SAE1 contains a single domain that adenylates SUMO and is homologous to the N-terminal portion of the ubiquitin E1 enzyme [5,14]. SAE2 is homologous to the C-terminal portion of the ubiquitin E1 enzyme and mediates exclusively the E1–SUMO interaction [5,15,16].

Through a transesterification reaction, activated SUMO is subsequently transferred to the catalytic cysteine of the unique SUMO conjugating (E2) enzyme, Ubc9 [17] which, in contrast to ubiquitin conjugating enzymes, has the ability to recognize target proteins directly and catalyze the formation of an isopeptide bond between the C-terminal glycine of SUMO and the ε-amino group of a target lysine [18]. Consistent with structural studies showing direct recognition of this consensus motif by the Ubc9 active site, recombinant E1, E2, and SUMO are sufficient for ATP-dependent SUMO modification of substrates *in vitro* [8,18]. 

SUMOylation is a dynamic and reversible process, and requires SENPs to remove SUMO conjugates from substrates, maintaining the reserve of the free SUMO form [8]. These proteins in general are able to play both roles in SUMO regulation: cleaving the isopeptide bond between SUMO and its substrate, and processing SUMO precursors to mature forms [5,18]. 

Despite the extensive proteomic analyses of SUMOylated proteins that have been conducted in several eukaryotic cells including parasites such as *Toxoplasma gondii* [19], *Plasmodium falciparum* [20]and *Trypanosoma* [21,22], the SUMOylation system of *Giardialamblia* has not been investigated until now. Previously, we demonstrated that the enzyme arginine deiminase is a SUMOylated protein [23] and this is so far the only evidence of SUMOylation in *Giardia*. Therefore, the identity, function, and regulation of SUMO proteins and the cellular processes they regulate are largely unknown. 

In the present work, we disclosed the presence of a single gene in *Giardia* (*gsumo*) that codifies to SUMO protein (gSUMO) and identifies gene encoding to putative enzymes of the SUMOylation pathway. To understand the role of SUMOylation in this parasite, we over-expressed gSUMO in transgenic trophozoites and produced monoclonal antibodies against recombinant SUMO protein (GST-gSUMO). These tools allowed us to reveal the presence of several putative SUMOylated proteins, suggesting SUMO conjugation as a functional system in *Giardia* and formerly in evolution.

## 2. Results and Discussion

### 2.1. Characterization of SUMO in Giardia lamblia

By searching in the GDB, we found that the *G. lamblia* genome encodes only one putative SUMO gene (ID GL7760). This matches what was reported for *Saccharomyces cerevisiae* [16,24,25]*,* invertebrates [5,18] and other parasites like *Plasmodium falciparum* [20], *Toxoplasma gondii* [19], *Trypanosoma brucei* [22,26] and *Trypanosoma cruzi* [21] but is dissimilar to what was found in *Arabidopsis thaliana* [27] and mammals [10,16], where multiple members of the SUMO family are present. ID GL7760 codes for a putative gSUMO protein of 102 amino acid residues, with a predicted molecular mass of 12 KDa. 

**Figure 2:**
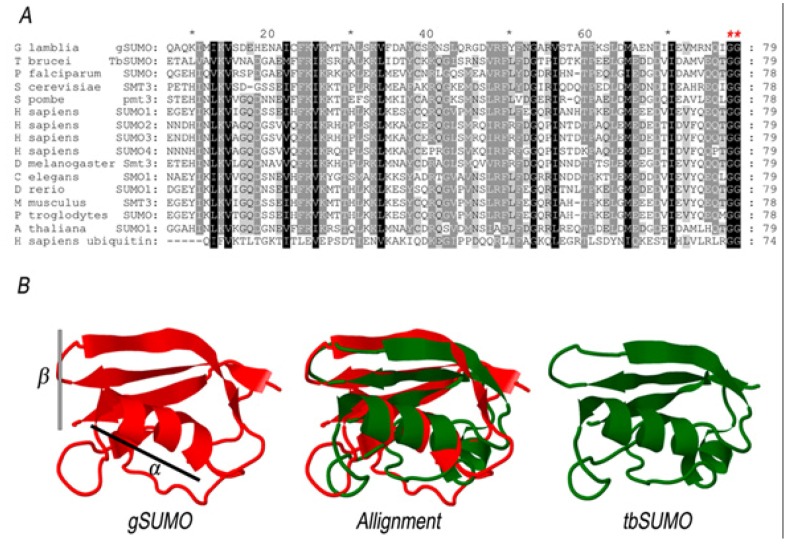
Comparative analysis of SUMO proteins. (**A**) Multiple sequence alignment was constructed between the conserved domain of gSUMO and other 13 SUMO sequences, plus Human Ubiquitin. Dark shading show identical residues, while light shading shows similar residues between the respective sequences. Red asterisks indicate conserved GG motifs. (**B**) Structural alignment was constructed with alfa-helix (α) and beta-sheet (β) portions of the predicted 3D structure of gSUMO and the Chain A of the Protein Data Bank file 2K8H from *T. brucei* (TbSUMO).

Multiple Sequence Alignment (MSA) of conserved regions of known SUMO family members, showed that gSUMO possesses the characteristic C-terminal region, which includes the conserved GG motif required for isopeptide bond formation [Figure 2(A)]. Sequence comparison also demonstrated that gSUMO is somehow homologous to the SUMO-1 member of the human SUMO family. The three-dimensional model of gSUMO was obtained using Phyre2, based on crystal structures of human SUMO-1 (PDB code 1A5R). Among the available PDB structures, human SUMO-1 was the one that shared higher sequence identity with gSUMO, revealing also the three-dimensional structure alignment of more conserved regions, high overlap between predicted gSUMO and the crystal structure of SUMO from *Trypanosoma brucei* (PDB code 2K8H) [Figure 2(B)]. All this suggests that SUMO is a highly conserved protein among all organisms. In order to characterize the SUMOylation process in *Giardia lamblia*, we produced WB-strain transgenic trophozoites that stably expressed SUMO, containing three hemagglutinin (HA) epitope sequences in the N-terminus (HA-gSUMO). Over-expression of HA-gSUMO in trophozoites under the constitutive tubulin promoter revealed, by immunofluorescence assays (IFA) in epifluorescence microscopy, mainly cytoplasmic localization but nuclear localization in some cells (data not shown).

**Figure 3:**
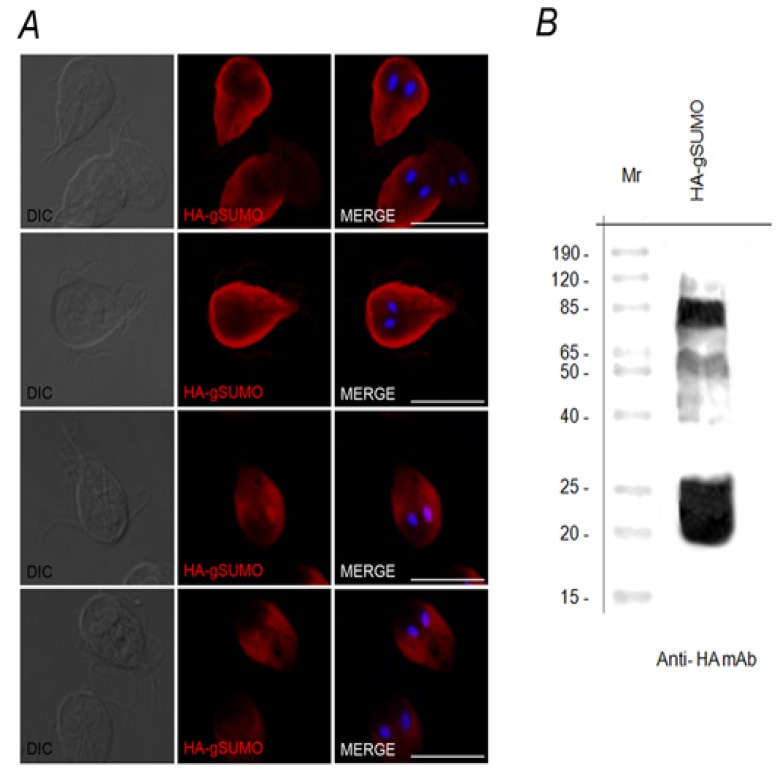
SUMO Over-expression. (A) HA-gSUMO localizes in both cytoplasm and nuclei in transgenic cells. IFA using anti-HA mAb and confocal microscopy shows that HA-gSUMO (red) has a variable pattern of localization from underneath plasma membrane and cytoplasm to nuclei. DIC (Differential Interference Contrast microscopy). Nuclei are stained with DAPI (blue). Scale bar: 10 µm. (B) Several putative SUMOylated substrates can be detected by Western blotting. Western blotting using anti-HA mAb shows a ~20 kDa band, likely corresponding to free SUMO form, plus bands that range from 50 kDa to 85 kDa that might correspond to SUMOylated substrates in HA-gSUMO transgenic trophozoites. Lane 1: Standars of the indicated molecular weights.

However, HA-gSUMO showed a more variable localization pattern (from underneath the plasma membrane to the nuclei) considering different focal planes of confocal microscopy (Figure 3A), which might suggest the presence of different putative substrates by SUMO. SUMOylation is known to target mainly nuclear proteins, although several studies point to many roles of SUMO in the soluble phase of the cytoplasm, the plasma membrane, mitochondria and the endoplasmic reticulum, documenting the presence of putatively SUMOylated proteins in both nucleus and cytoplasm, depending on the process being regulated [8,15,28].

Western blot analysis of HA-gSUMO transgenic trophozoites enabled the free SUMO form (~20 kDa), as well as many bands, to be observed (Figure 3B). Although SUMO has a molecular mass of approximately 11 kDa, it appears larger on SDS-PAGE and adds ~20 kDa to the apparent molecular weight of most substrates [29]. The other bands that range from ~50 kDa to 85 kDa might correspond to SUMOylated proteins. After cell fractionation and comparison with what we found in the IFA assays, these proteins were observed in both cytoplasmic and nuclear fractions (data not shown).

It is known that identification of SUMOylated proteins is not simple, for several reasons: (i) many SUMOylated proteins are present at a level below the normal detection limits [25,30], (ii) for most SUMO target proteins, only a small fraction of the substrate is SUMOylated at any given time, and (iii) there are strong SUMO protease activities in native cell lysates [25]. The list of newly discovered SUMO substrates is expanding only recently in other model systems, whereas only the arginine deiminase enzyme has been identified as a SUMOylated substrate in *G. lamblia* [23]. 

To analyze stage-specific dynamics of the protein SUMOylation in more detail, we generated a novel set of monoclonal antibodies against a recombinant GST–gSUMO protein. Mouse antisera against fusion protein were analyzed for specificity by dot-blot using GST–gSUMO, and IFA and dot-blot using total protein from wild-type trophozoites (data not shown). Mice showing the strongest positive reaction were later sacrificed and utilized to produce monoclonal antibodies (mAbs). Although several mAbs recognized the SUMO protein, we chose the clone 13C5, which showed the strongest reactivity in Western blot and in IFA assays.

**Figure 4:**
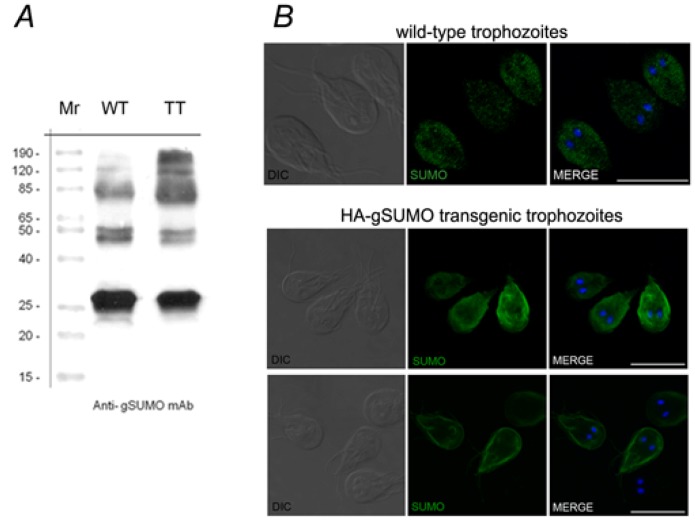
**gSUMO antibody reactivity.** (**A**) gSUMO mAb is able to recognize endogenous gSUMO. Western blotting of wild-type (WT) and HA-SUMO transgenic *Giardia* trophozoites (TT) show that the 13C5 mAb recognizes endogenous gSUMO (arrow) and several bands (from 50 kDa to 120 kDa) likely corresponding to putative SUMOylated protein. Lane 1: Standards of the indicated molecular weights. (**B**) IFA and confocal microscopy using the 13C5 mAb in permeabilized wild-type trophozoites showed a main pattern of gSUMO localization (green) in the cytoplasm while in and HA-gSUMO transgenic trophozoites the label is in the cytoplasm (botton panel) and concentrated close to the plasma membrane (upper panel). In CHO cells, a cytoplasmic localization is observed using the 13C5 mAb. Scale bar: 10 µm.

Western blot assays using homogenate of wild-type or gSUMO-transgenic trophozoites showed that this mAbs was able to recognize endogenous gSUMO in a similar pattern of bands to the one observed for transfected cells (Figure 4A). Similarly, IFA using the mAbs showed that the localization of gSUMO was cytoplasmic in wild-type, and concentrated around nuclei, plasma membrane and flagella in HA-gSUMO transgenic trophozoites. The difference of pattern localization between wild type and transgenic trophozoites is probably due to the increased expression of the protein in HA-gSUMO cells. Given the homology between gSUMO and SUMO-1, we analyzed in a eukaryotic CHO cell line the cross-reaction of the mAbs generated against the SUMO protein. We found a clear cytoplasmic subcellular localization, similar to the localization observed in *Giardia* wild-type trophozoites (Figure 4B). This result reinforces the feature of SUMO as a conserved protein in evolution. 

### 2.2. Identification of the SUMOylation Pathway Components

**Figure 5:**
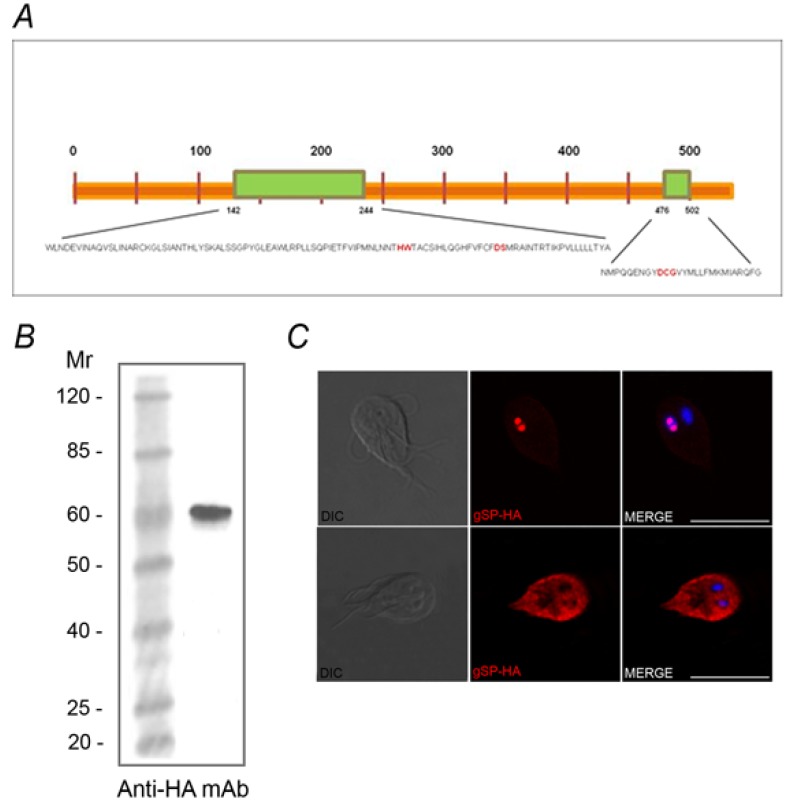
*Giardia lamblia* has one predicted gSP. (**A**) Schematic representation of the putative gSP containing two catalytic C-terminal domains (green) with the essential catalytic residues (red). (**B**) Western blotting showing a band of ~ 60 kDa (arrow) corresponding to free gSP in gSP-HA transgenic trophozoites (TT). (**C**) IFA using anti-HA mAb and confocal microscopy show gSP-HA (red) in the cytoplasm and nuclei (DAPI) of transfected *Giardia* trophozoites. WT: wild-type. Scale bar: 10 µm.

The SUMOylation pathway is a reversible process that creates an *on and off* state, which is essential for biological regulation. Besides the variation in *sumo gene* complexity in different species, the SUMOylation and de-SUMOylation components are conserved in most of the eukaryotes where this process was described [5]. Although the SUMOylation system appears to be a functional pathway for protein modification in *Giardia*, only the gene that encodes to putative gSUMO Protease (gSP: ID GL16438) protein, with a predicted molecular mass of 60 kDa was found in itsgenome. *In silico* analysis revealed the presence of a nuclear localization signal (NSL) *RPKR* at 333 position, and disclosed that the putative gSP might be a cysteine protease included in the C48 cysteine protease family, similar to other SUMO proteases described [5,31]. Although gSP presents low identity with other SUMO proteases characterized, it possesses two preserved C-terminal domains and contains a putative catalytic triad (histidine, aspartate, and cysteine), with a conserved glutamine residue essential for the formation of the oxyanion hole in the active site [31,32] (Figure 5A), suggesting that the essential catalytic residues are conserved. Over-expression of the C-terminus HA-tagged gSP (gSP-HA) was used to characterize gSP in growing trophozoites by IFA and Western blot (Figure 5B). Like gSUMO, gSP-HA localized in the cytoplasm, concentrated close to the plasma membrane including the flagella (Figure 5C) and showed nuclear localization in some trophozoites. 

All SUMO proteases possess a large N-terminal domain with minimal or no homology to each other’s domain. It has been suggested that the diversified N-terminal domains of these proteases determine their substrate specificity by controlling their subcellular localization [33,34,35]. Studies in both yeast and mammalian systems suggest that subcellular localization contributes to substrate selection by the SUMO proteases *in vivo* [36]. Distinct subcellular localization has been found by the mammalian SUMO proteases. Thereby, SENP1 has been localized to the nucleoplasm and nuclear bodies, SENP2 has been found at the nuclear pore, SENP3 localized to the nucleolus, and SENP6 was cytoplasmic [5,8,37]. 

Nevertheless, very little is known about the substrate specificity of each of the SUMO enzymes, and how a specific subcellular localization is linked to the function of each SUMO protease remains poorly understood. An interesting evidence was provided by Itahana *et al.* who observed a nucleocytoplasmic shuttling of human SENP2. Like SENP2 it is possible that gSP may have substrates in the nucleus and the cytoplasm showing similar pattern subcellular localization to gSUMO protein, and may the deSUMOylation of substrates be regulated by cellular processes, such as cell cycle progression. By controlling the function of the nucleocytoplasmic shuttling of gSP, cells could be able to selectively deSUMOylate specific substrates in the nucleus and/or in the cytoplasm, thereby achieving a greater flexibility in regulating gSP activity. Undoubtedly, future studies will reveal more about the functional specificity of the SUMO protease in *Giardia* and the relationship between the enzyme and the SUMO protein localization.

By searching the *G. lamblia* genome using the SAE1/2 sequence homologies, we identified one gene encoding putative gSAE1 (ID GL10661) and one gene that codified to putative gSAE2 (ID GL6288). Although putative gSAE1 is included by *in silico* analysis in the Ubiquitin E1 enzymes family protein it does not present the typical structure observed in other SAE1 isoforms characterized [38]. 

**Figure 6:**
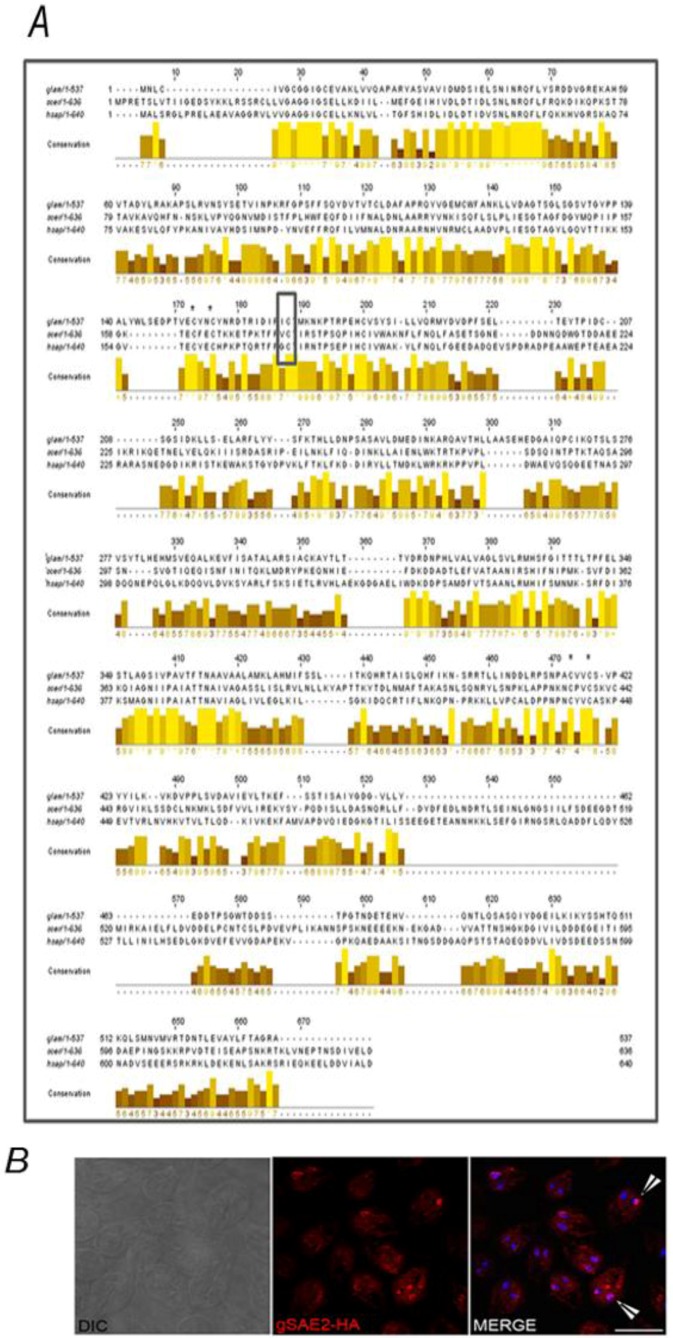
*In silico* analysis and localization of the putative gSAE2. (**A**) Jalview image shows amino acid sequence alignment of the SAE2 from *G.mlamblia* (*glam*), *S. cerevisiae* (*scer*), and *H. sapiens* (*hsap*). Bar graphs showing the amino acid conservation at each residue are shown. The putative active-site cysteine residue (C178 in *Giardia*) is indicated by empty black box while the cysteines corresponding to putative Zn^+^ motive are denoted by asterisks. (**B**) IFA using anti-HA mAb (red) and confocal microscopy show the cytoplasmic and nuclear (DAPI) localization of gSAE2-HA (arrow). Scale bar: 10 µm.

gSAE2 is a homologous protein to human SAE2 or yeast Ulp2p subunits and, similar to those, possesses a putative Zn^2+^ motif formed by Cys residues 152, 155, 416, and 419 [14] involved in direct binding of SUMO for adenylation, and three conserved domains that include the adenylation domain (ThiF family) (3–134), which binds both SUMO and ATP, the putative catalytic Cys domain (151–183) with the catalytic cysteine (putative C167 in *Giardia*) responsible for E1-SUMO-thioester bond formation [39], and the UbL or ubiquitin-like domain (315–378), due to its structural similarity to Ub and other Ubl modifiers [15] (Figure 6A). *In silico* analyses predicted a cytoplasmic localization for the putative gSAE2. IFA revealed a cytoplasmic localization but also nuclear and perinuclear (Figure 6B). 

**Figure 7:**
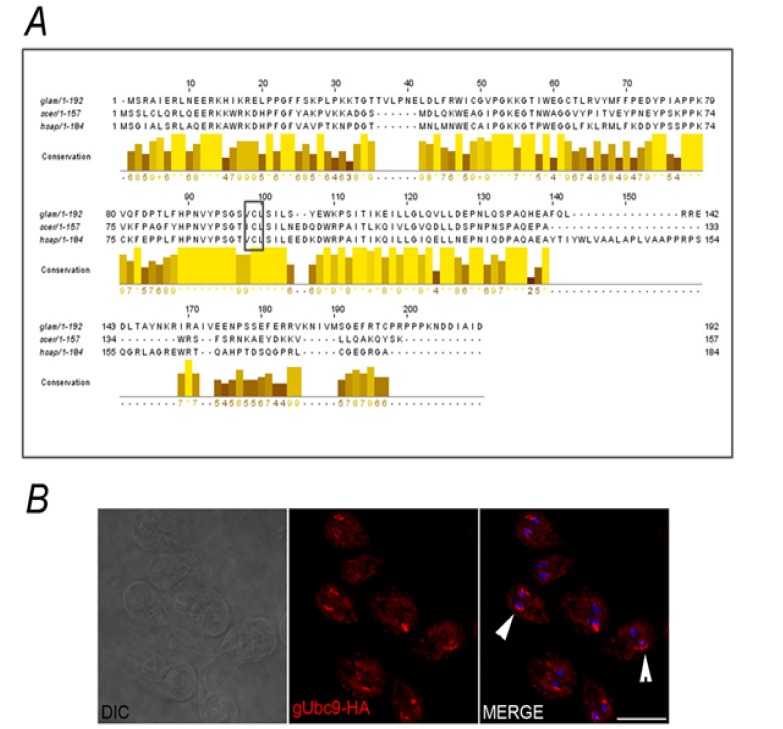
Putative gUbc9. (**A**) Alignment of putative gUbc9 with yeast and human Ubc9 orthologous shows high number of conserved amino acids residues and conserved putative cysteine (C98 in *Giardia*) required for thio-ester formation between SUMO and the Ubc9 enzyme (boxed and highlighted by a black bar). Bar graph shows the amino acid conservation at each residue. (**B**) IFA using anti-HA mAb (red) and confocal microscopy show that the enzyme possesses a cytoplasmic localization with the main distribution surrounding the nuclei (arrow) in gUbc9-HA transgenic trophozoites. Scale bar: 10 µm.

With bioinformatics tools, we also found one gene encoding by putative gUbc9 (ID GL24068) that, similar to other E2 family members, shares a conserved UbL domain of approximately 14 kDa and contains a conserved cysteine residue (putative C98 in *Giardia*) required for the thio-ester formation between SUMO and the E2 member (Figure 7A) [40]. *In silico* analyzing predicted a nuclear subcellular localization of putative gUbc9, similar to other SUMO-conjugating enzymes described [5]. However, IFA assays enabled us to observe that gUbc9 is present in the cytoplasm and surrounding the nuclei but not inside them (Figure 7B).

It is known that SUMO is conjugated to target proteins by an analogous but distinct pathway from ubiquitin conjugation [41], and that ubiquitin enzymes (specifically E1-activating and E2-conjugating enzymes) are highly related to the SUMOylating E1 and E2 enzymes [8]. In contrast to ubiquitin system where E3 ligase enzymes are generally a requirement for ubiquitination, in the SUMOylation pathway the requirement of E3 ligases is a source of debate because SUMO conjugation can be reconstituted under select conditions *in vitro* using only E1, Ubc9, SUMO, and ATP [16]. Although no putative E3-like ligase was found in the *Giardia* genome previous reports suggest that Ubc9 is sufficient in SUMOylation as long as the consensus sequence is present [18]. Nearly all SUMO-modified proteins identified to date contain a conserved motif that surrounds the modified lysine, and structural and mutational analyses of this motif indicate that it is recognized directly by Ubc9. Thus, the direct interaction between Ubc9 and SUMO substrates seems to preclude an absolute requirement for E3-like factors [42]. Also, *Giardia*’s genome encodes a simplified form of many cellular processes: fewer and more basic subunits, incorporation of single-domain bacterial and archaea-like enzymes, and a limited metabolic repertoire that makes some proteins functionally redundant with other proteins in the same or another pathway. Therefore, it is possible that *Giardia* has acquired a basal protein SUMOylation system based on unusual features compared to other organisms [4]. 

## 3. Experimental Section

### 3.1. Parasites, Cells and Media

*G. lamblia* trophozoites of the isolate WB, clone 1267 (WB/1267) were axenically cultivated in screw-cap borosilicate glass tubes in modified TYI-S-33 medium enriched with 10% heat-inactivated fetal bovine serum at pH 7.5 and supplemented with 0.1% bovine bile [43]. Trophozoites of the WB/1267 clone were transfected by electroporation and selected with puromycin [44,45]. The transfection of stable WB/1267 cells was 100%, as determined by IFA and flow cytometry. Cultures were harvested by chilling on ice followed by agitation to dislodge attached cells. Trophozoites were collected by centrifugation at 500 × g for 10 min at 4°C and washed three times with PBS. 

The mouse myeloma cell line NSO (ECACC85110503) was grown in RPMI 1640 (GIBCO) supplemented with 10% fetal bovine serum.Chinese hamster ovary (CHO) cells were grown to confluence in D-MEM (GIBCO) medium supplemented with 10% fetal bovine serum. The cells were maintained in a humidified incubator at 37 °C with 5% CO_2_.

### 3.2. Mice

Purebred female BALB/c mice (aged 10–12 weeks) were purchased from the Facultad de Ciencias Veterinarias, Universidad de La Plata, and housed at the vivarium of the Instituto Mercedes & Martín Ferreyra (INIMEC-CONICET). They were maintained in our animal facilities, which meet the conditions of the Guide to the Care and Use of Experimental Animals, published by the Canadian Council on Animal Care (with the assurance number A5802-01 being assigned by the Office of Laboratory Animal Welfare (NIH)). Our Institutional Experimentation Animal Committee also approved the animal handling and experimental procedures.

### 3.3. Bioinformatics Analysis

Searching in the *Giardia* genome data base (GDB), only one SUMO homologue (gSUMO, ID GL7760) was identified using the human SUMO-1 sequence as a query. In order to test the conservation of the amino acid composition, a multiple sequence alignment was performed using T-Coffee [46] with default settings and SUMO sequences from *H. sapiens*, *P. troglodytes*, *M. musculus*, *D. rerio*, *D. melanogaster*, *C. elegans*, *S. cerevisiae*, *S. pombe*, *T. brucei*, P*. falciparum* and *A. thaliana*. Following the alignment, Block Mapping and Gathering with Entropy software [47] was used to select regions of the alignment with a higher percentage of identity. The resulting shorter alignment (79 residues on average, between positions 34 and 111) was then manually curated with GeneDoc [48]. 3D structure of gSUMO was predicted using Phyre2 [49]. Alfa-helix and Beta-sheet regions (between positions 23 and 96) corresponding to gSUMO and Chain A of the Protein Data Bank file 2K8H from *T. brucei* [22] were aligned with the program Friend 2.0 [50].

Genes coding for putative Ubiquitin-like proteins in *G. lamblia* were obtained from GiardiaDB. To identify the putative components of the SUMOylation pathway, previously characterized yeast and human orthologues were obtained from NCBI (http://www.ncbi.nlm.nih.gov/BLAST/) and used as query on gBLASTp. Multiple sequence alignments were constructed using Muscle and Jalview algorithm. PSORT (http://psort.hgc.jp/) and phobius (http://phobius.sbc.su.se/) were used to *in silico* analysis of proteins. 

### 3.4. Isolation of *gsumo*; gsp; *gsae2*; *gubc9*

Genomic DNA was prepared from *G. lamblia* as described earlier and used as a template to amplify the *gsumo* gene with the primers gsumoF and gsumoR (forward primer: 5'- CATTGGATCGGATGACGAAGGAGACGTCCCCCCAATT-3'; reverse primer: 5'-CATTGCGG CCGCCTAGTGGCCGCCAATCTGATTTCGCATCA-3') containing a BamH1 and Not1 restriction enzyme site, respectively. To amplify the *gsp* gene we used the primers gSPF and gSPR (forward primer: 5’- CATTCCATGGCTGCTGAACTGTTGCAGCTCAAA-3’; reverse primer: 5’-CATTGAT ATCGCAGAGCTCGTCCAGATCTTGCGG-3’) containing a NcoI and EcoRV restriction enzyme site. To amplify the *gsae2* gene we used the primers: gsae2F 5’-CATTCCATGGATCTGTGCATCGT CGGGTGCGGC-3’ and gsa2R: 5’-CATTGATATCGCAGAGCTCGTCCAGATCTTGCGG-3’ containing a NcoI and EcoRV restriction enzyme site. To amplify the *gubc9* gene we used the primers gubc9F and gubc9R (forward primer: 5’-CATTCCATGGAATTGGCTTTTAAAACACGAAATTCA GTTAAAATGG -3’; reverse primer: 5’- CATTGATATCCTTTTTGCTGGCGTAGTCAAGCGT-3’) containing a NcoI and EcoRV restriction enzyme site. PCR reactions were carried out in a thermal cycler (Eppendorff, Germany) with denaturation at 94 °C for 2 min before 30 cycles of 94 °C for 1 min, 57 °C for 30 s, 72 °C for 1 min 30 s and 72 °C for 7 min for final extension. PCR products were purified (QIAquick PCR purification kit, Qiagen) and digested with BamH1 and Not1 enzymes or NcoI and EcoRV enzymes respectively. 

### 3.5. Plasmid Construction by gSUMO Over-expression

Standard recombinant DNA techniques were used to construct the plasmid gSUMO-ptubHA and ptubApaHA-gSP, ptubApaHA-gSAE2, and ptubApaHA-gUbc9. Fragments of 309 bp corresponding to gsumo, and 1620 bp, 1614 bp and 538 bp corresponding to the putative *gsp* gene, putative *gsae2* gene and *gubc9* gene respectively, were generated by PCR amplification of genomic DNA. PCR products were purified (QIAquick PCR purification kit, Qiagen), then digested with BamHI-NotI, and NcoI-EcoRV ligated in frame with HA tag sequence at the N-terminus of ptubHA expression vector or with HA tag sequence at the C-terminus of ptubApaHA expression vector with T4 DNA ligase (MBI fermentas). Plasmids were introduced into *E. coli* X-10 Gold by CaCl_2_ transformation and the insert was confirmed by colony PCR and DNA sequencing (Macrogen, South Korea).

### 3.6. Recombinant Protein gSUMO Fused to GST Expression and Purification

Production of the recombinant protein GST-gSUMO has been described in detail previously [51]. Briefly, cDNA encoding *Giardia* SUMO was amplified and cloned into the gluthatione-S-transferase (GST) fusion expression vector, pGEX-4T 3 (Amersham Pharmacia Biotech, Little Chalfont, United Kingdom) via BamHI and NotI restriction sites. The GST-tagged protein was expressed in Escherichia coli strain BL21-Codon-Plus (Stratagene, Valencia, CA) and purified using Gluthatione-Sepharose 4B beads (Amersham Pharmacia Biotech) yielding amounts sufficient for mouse immunization.

The eluted recombinant gSUMO protein fractions were separated on 12% SDS-PAGE and visualized by Coomassie blue staining.

### 3.7. Monoclonal Antibody Production

The recombinant protein GST-gSUMO was used as antigen for mouse immunization and monoclonal antibody production. Three female BALB/c mice were subcutaneously injected with 100 μg of antigen emulsified with TiterMax Gold Adjuvant (Sigma, St. Louis, MO) (1:1) on days 1 and 15. On day 30, mice were boosted intravenously with 100 μg of the antigen in PBS. The mouse myeloma cell line NSO was used for fusion with spleen cells obtained from immunized mice. Antibody secreting hybridomas were screened by indirect immunofluorescence and dot-blotting, using non-encysting WB trophozoites, and were then grown, screened and finally cloned.

### 3.8. Western Blotting

For Western blot assays, parasite lysates were incubated with sample buffer with b-mercaptoethanol, boiled for 10 min, and separated in 10% Bis-Tris gels using a Mini Protean II electrophoresis unit (Bio-Rad). We used 50 µg and 500 µg of proteins for wild type or transgenic trophozoites, respectively. Samples were transferred to nitrocellulose membranes, blocked with 5% skimmed milk and 0.1% Tween 20 in TBS, and then incubated with hybridoma supernatants (1:200) or anti-HA monoclonal antibody (Sigma) for an hour. After washing 3 times with 0.1% Tween 20 in TBS, the strips were incubated for 1 h with horseradish peroxidase-conjugated polyclonal goat anti-mouse Igs (Dako) and then visualized with autoradiography. Controls included the omission of the primary antibody and the use of an unrelated antibody.

### 3.9. Immunofluorescence Assay

Trophozoites cultured in growth medium were harvested and processed as described. Briefly, cells were washed with PBSm (1% growth medium in PBS, pH 7.4), allowed to attach to multi-well slides in a humidified chamber at 37 °C for an hour, and the wells were fixed for 40 min with fresh 4% formaldehyde. The cells were incubated sequentially with blocking solution (10% goat serum and 0.1% triton X-100 in PBS) at 37 °C for 30 minutes followed by incubation with anti-HA mAb (1/300) or undiluted hybridoma supernatant at 37 °C for an hour. After washing three times with PBS, the cells were incubated for 1 h in the dark with FITC-conjugated goat anti-mouse secondary antibody (Cappel, Laboratories) or Texas red anti-mouse secondary antibody. Finally, preparations were washed and mounted in Vectashield mounting media. Fluorescence staining was visualized by using a conventional (Zeiss Pascal) inverted confocal microscope, using 100× oil immersion objectives (NA 1.32, zoom X). Differential interference contrast images were collected simultaneously with fluorescence images by the use of a transmitted light detector. Images were processed using FV10-ASW 1.4 Viewer and Adobe Photoshop 8.0 (Adobe Systems) software.

## 4. Conclusions

In this work we present evidence of SUMOylation in *G. lamblia,* a protozoan parasiteconsidered a basal organism in eukaryotic evolutionary history. By searching the GiardiaDB, we identified a SUMO gene with products that are highly homologous to SUMO-1 isoforms and SMT3C of yeast, a gene that encodes for a putative gSP, and two proteins, putative gSAE2 and putative Ubc9, with high identity and homology to the SUMOylation enzymes, presumed to function in both the SUMOylation and the ubiquitination pathways [5,8,52]. The over-expression of SUMO in *Giardia* and in the mAbs produced, enabled us to describe the localization of SUMO in wild-type and transgenic trophozoites and the presence of potential SUMO conjugates. However, research about how SUMO affects biological processes is only in its early stages. Knowledge of the proteins targeted by this modification is of the utmost importance in deciphering the impact of SUMOylation on the biology of the organism. Experiments on SUMOylated candidates are currently underway and will help us to disclose how SUMOylation is regulated, how the SUMOylation process functions to integrate signal pathway networks, and the role of this post-translational modification in the *G.lamblia* life cycle and in the evolution of the eukaryotes.
